# Review of an innovative approach to practical trials: the ‘cohort multiple RCT’ design

**DOI:** 10.1186/1745-6215-16-S2-P114

**Published:** 2015-11-16

**Authors:** Clare Relton, Kate Thomas, Jon Nicholl, Rudolf Uher

**Affiliations:** 1 University of Sheffield, Sheffield, UK; 2 Dalhousie University, Halifax, Nova Scotia, Canada

## Background

The ‘cohort multiple randomised controlled trial’ (cmRCT) is an innovative approach to the design and conduct of RCTs which compare the effectiveness of interventions to usual care (Relton et al, 2010). The design utilises a large long term observational cohort of people with the condition of interest, regularly measuring the outcomes of the whole cohort. The cohort in the cmRCT design allows multiple trial populations to be quickly identified and recruited and interventions tested against usual care. Information consent processes are similar to those in routine healthcare.

## Methods

Studies using the design were identified through citations of the original theoretical article (Relton et al 2010). Data were extracted from published articles, study protocols and presentations.

## Results

16 studies implementing the cmRCT design were identified with a total of 18 ongoing or completed trials were embedded within these cohorts. Some cohorts focussed on a single disease or injury (e.g. hip fracture, breast cancer, colorectal cancer), others had a wider focus (e.g. risk of mental health conditions, risk of falls). Some studies built a cohort around a trial, and then obtained further funds to exploit the cohort for further trials within that cohort.

## Conclusions

This review of the cmRCT design in practice provides examples of the design in the UK, Canada and the Netherlands and will help guide researchers interested in using the cmRCT design. Future research needs to assess the acceptability and efficiency of this approach, i.e., if/when this design is preferable to the standard approach to single separate RCTs.

